# *Xylaria insolita* and *X. subescharoidea*: two newly described species collected from a termite nesting site in Hua-lien, Taiwan

**DOI:** 10.1186/s40529-020-00287-1

**Published:** 2020-04-06

**Authors:** Huei-Mei Hsieh, Jyh-Ching Chou, Yu-Ming Ju

**Affiliations:** 1 Institute of Plant and Microbial Biology, Academia Sinica, Taipei, 11529 Taiwan; 2 Department of Natural Resources and Environmental Studies, National Dong Hwa University, Hualien, 97401 Taiwan

**Keywords:** Ascomycota, Phylogeny, Taxonomy, Xylariaceae

## Abstract

**Background:**

A number of *Xylaria* species are exclusively associated with nests of macrotermitine termites. A nesting site of *Odontotermes formosanus* in eastern Taiwan, which is the only macrotermitine termite known on the island, had been inundated during the raining season of 2010, and hundreds of *Xylaria* stromata emerged from it thereafter. A thorough examination of these stromata showed that they represent a mixture of different species.

**Results:**

Five *Xylaria* species were identified from the stromata collected from the nesting site, including two undescribed species, which are newly described as *X. insolita* and *X. subescharoidea* herein, and three known species *X. brunneovinosa*, *X. escharoidea*, and *X. furcata.*

**Conclusion:**

Totally, there are 28 *Xylaria* species growing on termite nests or ground in the world. Although *O. formosanus* is the only macrotermitine species known in Taiwan, the *Xylaria* diversity associated with its nests is fairly high; the species number has reached 12 with *X*. *furcata*, *X. insolita*, and *X. subescharoidea* added to the Taiwan mycobiota.

## Background

*Xylaria* species are characterized by upright massive stromata and high ascal apical rings, and geniculosporium-like anamorphs. More than 20 *Xylaria* species are specifically associated with fungus gardens built within nests of macrotermitine termites (Ju and Hsieh 2007; Rogers et al. 2005). There is only one macrotermitine species *Odontotermes formosanus* Shiraki found in Taiwan (Hsieh et al. 2017), and nine *Xylaria* species have been collected from its nests (Ju and Hsieh 2007).

In 2010 we conducted a survey on *Xylaria* species at a backyard of a residence in Hua-lien located in eastern Taiwan, where a nesting site of *O*. *formosanus* had previously been inundated following a heavy rain, and numerous *Xylaria* stromata kept emerging from the nesting site after termite activities had ceased. Among these stromata, several *Xylaria* species were identified, and two of these are undescribed species.

In this study, we describe the two undescribed species as new, i.e., *X. insolita* and *X. subescharoidea*. Their ITS, *β*-*tubulin*, *RPB2*, and *α*-*actin* were sequenced and analyzed in the context of the dataset mainly from Hsieh et al. (2010) to infer its phylogenetic relationships within *Xylaria*. *Xylaria* diversity emerging from a macrotermitine termite nesting site has poorly been documented, and we thus take the opportunity to comment on the *Xylaria* species found at the Hua-lien termite nesting site.

## Methods

### Collecting, fungal observation, isolation, and culturing

Species of *Xylaria* emerging from a nesting site of black-winged subterranean termite were surveyed from the backyard of a residence in Fu-hsin Village, Ji-an Township, Hua-lien County (23° 58′ 13.9″ N, 121° 33′ 14.5″ E) located in eastern Taiwan during June and September, 2010. Stromata were photographed on site, and collected stromata were air-dried after culture isolation.

Material was mounted in water and Melzer’s iodine reagent for examination of microscopic features by differential interference contrast microscopy and bright field microscopy.

Cultures were obtained by placing tissue from freshly collected stromata on SME medium (Kenerley and Rogers 1976). Resulting colonies were transferred to 9-cm plastic Petri dishes containing 2% Difco oatmeal agar (OA), from which the culture descriptions were made, and were incubated at 20 °C under 12 h fluorescent light.

### DNA extraction, PCR, cloning, and sequencing

ITS, *β*-*tub*, *α*-*act*, and *rpb2* are four loci commonly sequenced for inferring relatedness for xylariaceous fungi (Hsieh et al. 2010; U’Ren et al. 2016). PCR amplifications of *β*-*tub* and *α*-*act* were described in Hsieh et al. (2005), whereas those of *rpb2* and ITS were in Hsieh et al. (2009), (2010), respectively.

### Phylogenetic analyses

Dataset of concatenated sequences of *β*-*tub*, *α*-*act*, and *rpb2* was subjected to phylogenetic analyses. See Hsieh et al. (2010) for phylogenetic analyses, where Bayesian (BA) analyses and maximum parsimony (MP) analyses were performed with MrBayes 3.0b4 (Huelsenbeck and Ronquist 2003) and PAUP* 4.0b10 (Swofford 2003), respectively.

The combined sequences of *rpb2*, *β*-*tub* and *α*-*act* of *X. insolita* and *X*. *subescharoidea* were added to the RPB2-TUB-ACT dataset in Hsieh et al. (2010), with *X. coprinicola* Y.-M. Ju, H.-M. Hsieh and X.-S. He (Ju et al. 2011) and *X. terricola* Y.-M. Ju, H.-M. Hsieh and W.-N. Chou (Chou et al. 2017) also added. It should be noted that *X*. sp. 7 and *X*. sp. 8 in Hsieh et al. (2010) were later described as *X*. *reevesiae* Y.-M. Ju, J. D. Rogers and H.-M. Hsieh and *X*. *vivantii* Y.-M. Ju, J. D. Rogers, J. Fournier and H.-M. Hsieh, respectively, by Ju et al. (2018); *X. montagnei* Hamme and Guerrero is substituted by *X. cuneata* C. G. Lloyd (Ju et al. 2016); and *X*. cf. *glebulosa* (Ces.) Y.-M. Ju and J. D. Rogers is substituted by *X. rhytidosperma* J. Fourn. and Lechat (Fournier et al. 2018). Also, *Xylaria cubensis* (Mont.) Fr. and *X*. *laevis* C. G. Lloyd in Hsieh et al. (2010) are replaced by *X. flabelliformis (*Schwein.) Fr. and *X*. *cubensis*, respectively (Ju et al. 2016). The resulting dataset contained 135 isolates of 117 taxa (Additional file 1: Table S1), where major genera of the subfamily Xylarioideae as well as representatives of various groups and species aggregates of *Xylaria* were included. Three out-group taxa were *Annulohypoxylon cohaerens* (Pers.) Y.-M. Ju, J. D. Rogers and H.-M. Hsieh, *Biscogniauxia arima* San Martín, Y.-M. Ju and J. D. Rogers, and *B. mediterranea* (De Not.) Kuntze of the subfamily Hypoxyloideae.

## Results

### Survey of the collecting site

**Fig. 1:**
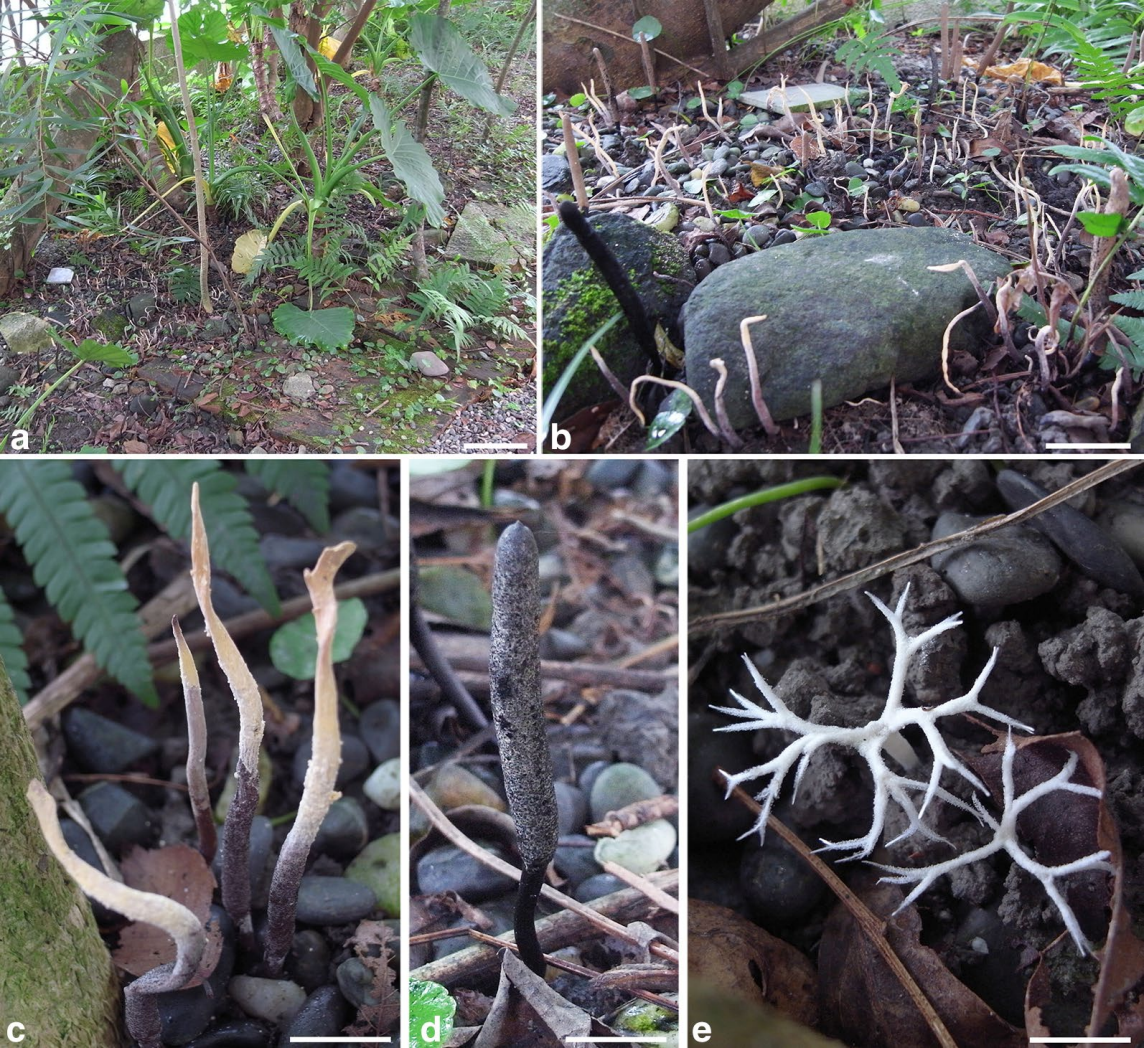
Collecting site, *X. brunneovinosa*, *X. escharoidea*, and *X. furcata*. **a**, **b** Collecting site. **a** Overall view. **b** Area where stromata are most populated. **c** Stromata of *X*. *brunneovinosa* with the upper portion still overlain with luteous conidial masses. **d** Stroma of *X*. *escharoidea*. **e** Immature stromata of *X*. *furcata*. Bars in **a** = 10 cm; **b** = 2 cm; **c**, **d** = 1 cm; **e** = 5 mm

More than 100 stromata were found at the collecting site (Fig. 1a, b). Totally, five *Xylaria* species were identified, including *X. brunneovinosa* Y.-M. Ju and H.-M. Hsieh (Fig. 1c), *X. escharoidea* (Berk.) Sacc. (Fig. 1d), and anamorphic *X. furcata* Fr. (Fig 1e), and two undescribed species, which are described herein as *X*. *insolita* and *X*. *subescharoidea*. Stromata of these five *Xylaria* species were intermixed with one another, not being restricted to particular areas at the nesting site. Most of the stromata were of *X. brunneovinosa* and *X. escharoidea*, and only 14 stromata of *X. insolita*, nine of *X. subescharoidea*, and two of *X. furcata* were found.

### Phylogenic analyses

**Fig. 2:**
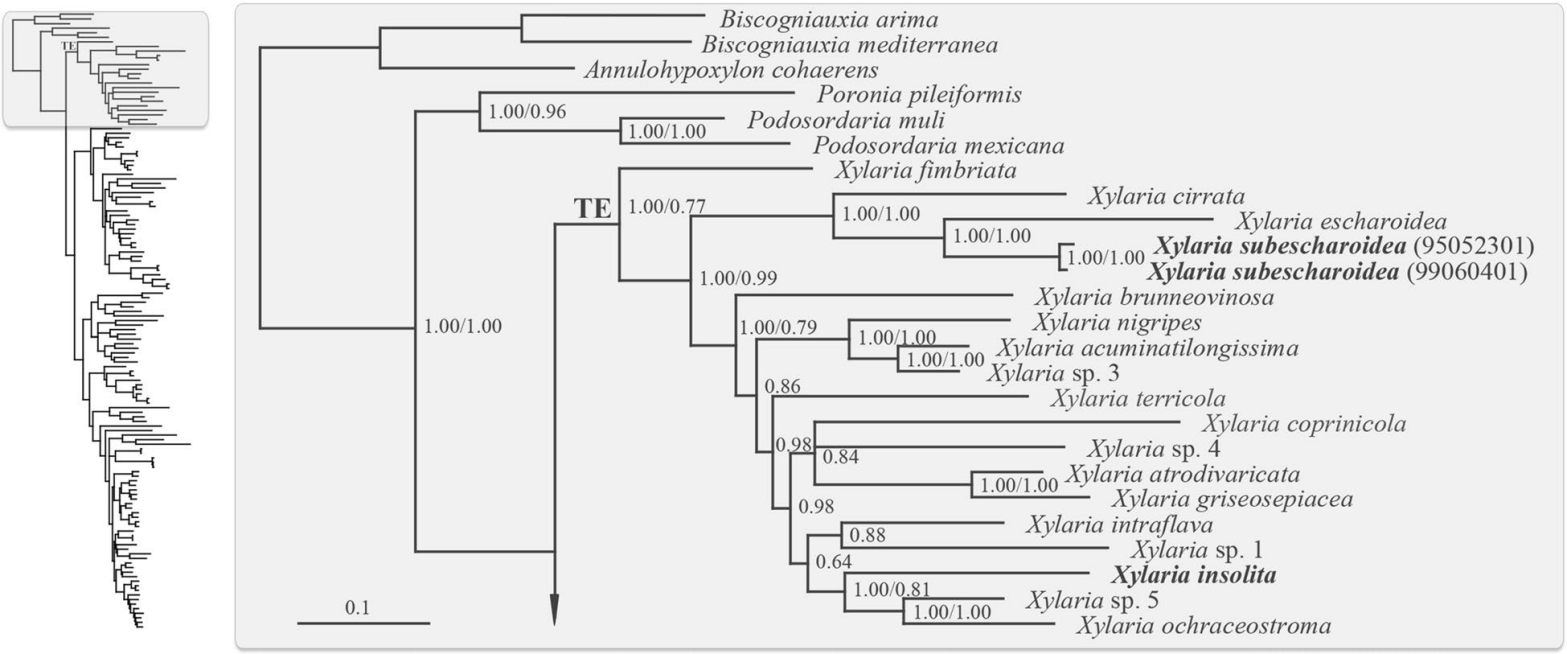
Phylogenetic tree generated by BA analysis from the RPB2-TUB-ACT dataset. The complete tree is shown schematically, with the area in the rectangle enlarged to show the detail of the clade denoted by TE, which encompasses *X. insolita*, *X. subescharoidea*, and *Xylaria* species associated with termite nests or soil. Numbers at internodes represent posterior probability values of a 50% majority rule consensus tree from a 1,000,000 generation Markov chain Monte Carlo analysis. These are immediately followed by the bootstrap values greater than 50%

With *X*. *insolita* and *X. subescharoidea* included in the phylogenetic analyses, the overall tree topologies resulting from BA (Additional file 2) and MP analyses were highly similar to those in Hsieh et al. (2010), having the two species grouped within the TE clade (Fig. 2), to which all of the studied *Xylaria* species of the subgenus *Pseudoxylaria* belong. We present only the portion of the tree concerning subgenus *Pseudoxylaria* in Fig. 2, which showed *X*. *insolita* clustering with *X*. sp. 5 and *X*. *ochraceostroma* Y.-M. Ju and H.-M. Hsieh and *X*. *subescharoidea* clustering with *X. escharoidea*.

### Taxonomy

**Fig. 3:**
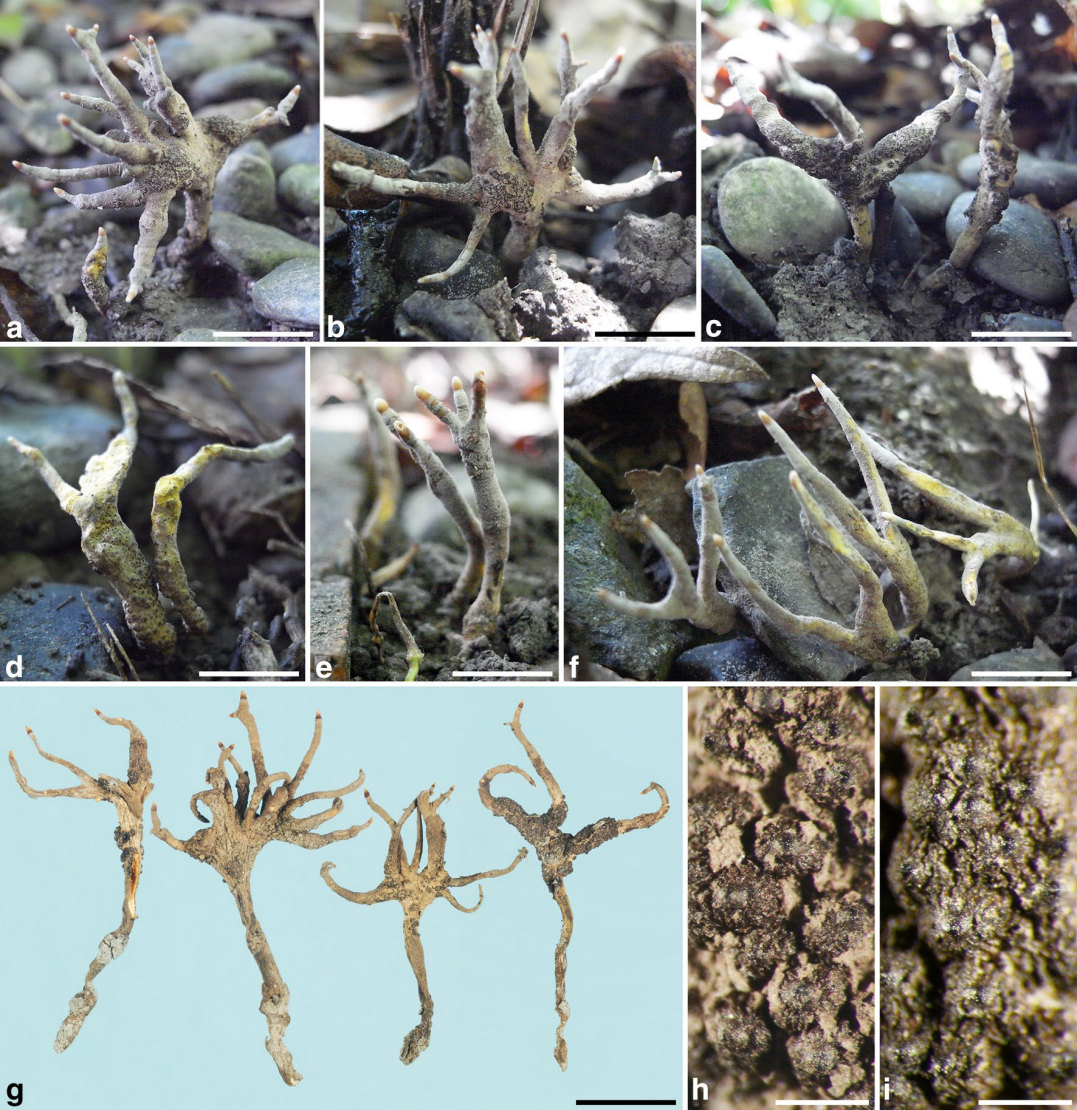
*Xylaria insolita* (from the holotype). **a**–**f** Stromata in natural habit; stromata in **e**, **f** are immature, bearing conidia only. **g** Dried stromata. **h** Stromatal surface with outer stromatal layer ruptured by developing perithecia into flaky remnants. I. Outer stromatal layer worn off to reveal the rugulose surface. Bars in **a**–**f** = 1 cm; **h**, **i** = 0.5 mm

**Fig. 4:**
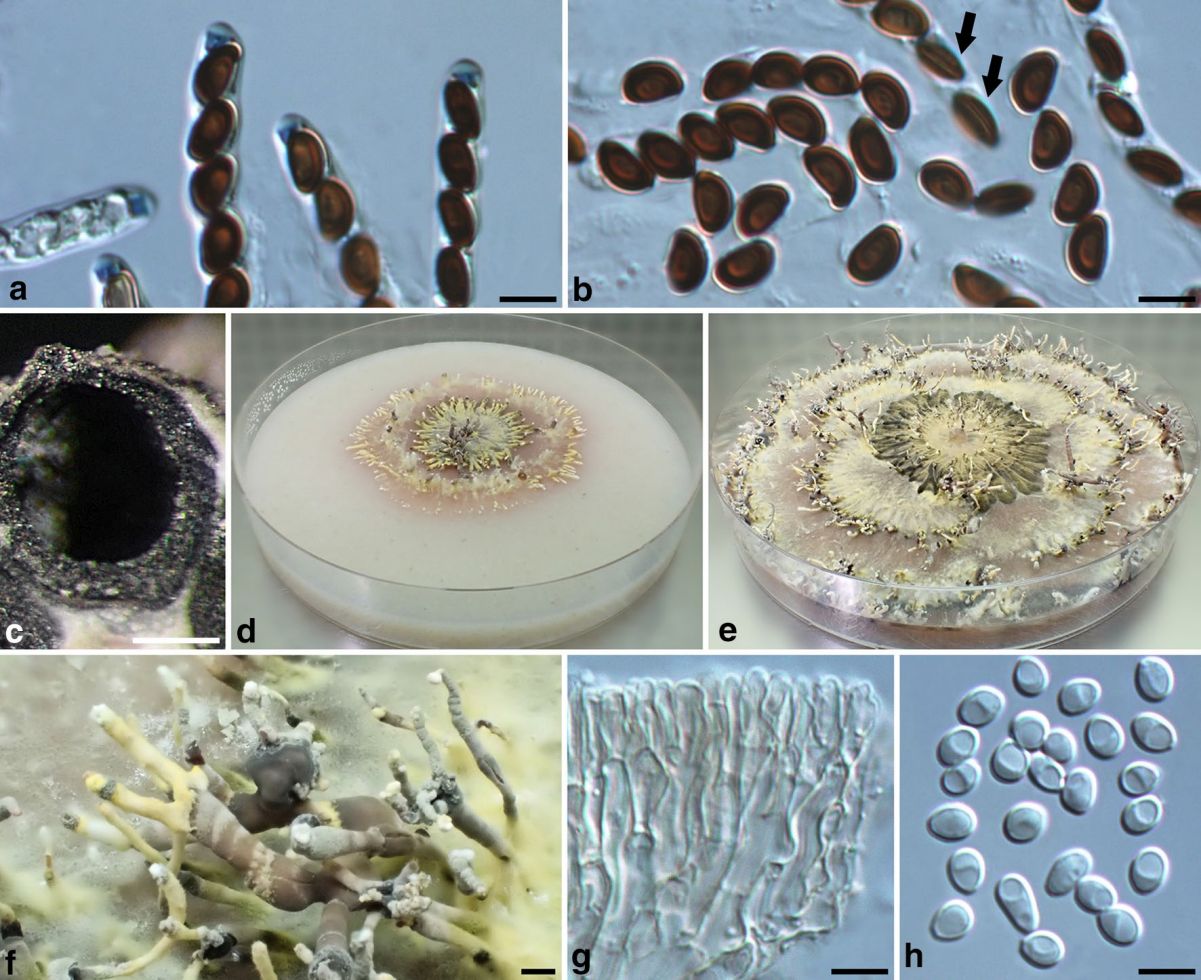
*Xylaria insolita* (from the holotype). **a** Ascal apical rings and ascospores. **b** Ascospores; the arrows point towards two ascospores showing a germ slit. **c** Vertical section of a perithecium. **d**, **e** Colony on 9-cm Petri plate containing OA at 2.5 week and 6 week, respectively. **f** Stromata produced in culture. **g** Conidiophores. **h** Conidia. Bars in **a**, **b**, **g**, **h** = 5 μm; **c** = 0.125 mm; **f** = 1 mm

***Xylaria insolita*** Y.-M. Ju, H.-M. Hsieh et J.-C. Chou, sp. nov. Figs. 3, 4.

MycoBank MB 834498.

**Etymology.** Denoting the highly variable palmate stromata.

Stromata palmate at fertile part, 2–15-digitate, with tapering sterile apices, substipitate or sessile, 2–4 cm in total length above ground, 1–2.5 mm diam at clavae; surface dull grayish brown with a yellow tinge, rugulose, with conspicuous to half-exposed perithecial mounds unevenly aggregated or evenly distributed, overlain with a grayish brown outer layer gradually ruptured by perithecial mounds into flaky remnants and sloughing off afterwards, underlain with a thin, soft, black layer ca. 10 µm thick; interior white, soft, homogeneous. Perithecia spherical, 300–400 µm diam. Ostioles coarsely conic-papillate, ca. 100 µm broad at base. Asci with eight ascospores arranged in uniseriate manner, cylindrical, 105–135 µm total length, the spore-bearing part 40–50 µm long × 4–5 µm broad, with an apical ring staining blue in Melzer’s iodine reagent, inverted hat-shaped, 1.6–2.2 µm high × 1.6–2.5 µm broad. Ascospores brown to dark brown, unicellular, ellipsoid-inequilateral, laterally compressed, with one end narrowly rounded and slightly beaked and the other end broadly rounded, smooth, (5.2–)5.6–6.2 (–6.7) ×  (3.3–)3.5–3.9 (–4.0) ×  (2.5–)2.6–2.8 (–3.0) µm (5.9 ± 0.3 × 3.7 ± 0.2 × 2.7 ± 0.1 µm, N = 40), with a straight germ slit spore-length or nearly so on the dorsal side, lacking a hyaline sheath; epispore smooth.

**Cultures and anamorph.** Colonies reaching the edge of 9-cm Petri dish in 5 week, yellowish, slightly cottony, zonate, with diffuse margins. Reverse fawn-colored. Stromata arising from concentric zones and strongly inclined outwards, cylindrical, tapering at top, unbranched or branched, 0.3–1.3 cm long × 0.5–1.2 mm diam, yellow grading to brown towards the base, white on the surface of upper part but becoming pale olivaceous gray due to production of conidia. Conidiophores in upright, densely arranged palisades, dichotomously branched several times from base, smooth, hyaline, grading to light brown downwards. Conidiogenous cells terminal, cylindrical, 6.5–12 × 2–3 µm, smooth, bearing terminal, slightly denticulate conidial secession scars. Conidia produced holoblastically in sympodial sequence, hyaline, smooth, obovoid to ellipsoid, (3.0–)3.5–4.7 (–6.4) ×  (2.5–)2.7–3.1 (–3.8) µm (4.1 ± 0.6 × 2.9 ± 0.2 µm, N = 40), with a flattened base indicating former point of attachment to conidiogenous cell.

**Typification.** TAIWAN. Hua-lien County, Ji-an Township, Fu-hsin Village, from termite nests underground, 3 Sep 2010, *Chou, J.*-*C. 99090301* (cultured) (holotype HAST 144970), GenBank accessions: ITS = MN655979, *rpb2* = MN656981, *β*-*tub* = MN656983, *α*-*act* = MN656985.

**Notes.***Xylaria insolita* is peculiar among *Xylaria* species in having highly variable palmate stromata and laterally compressed, slightly beaked ascospores with the germ slit on the dorsal side. Unlike most of the *Xylaria* species where the teleomorph and anamorph are produced in different times or on different stromata, *X. insolita* can have the anamorph and teleomorph coexist on the same stromata at the same time, with mature perithecia produced at the lower part of stromata and conidiogenesis on the finger-like terminals. Perithecial contours are conspicuous to half-exposed, evenly distributed or unevenly clumped together. The outer stromatal layer is ruptured by developing perithecia into flaky remnants, which remain attached at maturity but are gradually worn off afterwards.

Colonies on OA are yellowish, with stromata produced in concentric zones. The stromata produced in cultures never reach maturity, having a yellow surface and producing pale olivaceous gray conidial masses and resembling much those immature stromata produced in nature.

Phylogenetic analyses clustered *X*. *insolita* together with *X*. *ochraceostroma* and *X*. sp. 5, a fungus known only in anamorph. Unlike *X*. *insolita* where the conidiophores are in densely arranged palisades, *X. ochraceostroma* has repeatedly dichotomously branched conidiophores that arise singly on the stromatal surface and render the surface a granular appearance (Ju and Hsieh 2007). The general appearance of the conidiophores of *X*. *ochraceostroma* resembles that of terverticillate penicilli characteristic of *Penicillium* Link subgenus *Penicillium*. *Xylaria ochraceostroma* also differs from *X*. *insolita* by lacking a black layer beneath the ochraceous stromatal surface and having the ascospore germ slit on the ventral side. Conidiophores of *X*. sp. 5 also arise singly and have a swollen top, thus resembling the vesiculate conidiophores of *Aspergillus* P. Micheli ex Haller (unpublished data of Y-MJ).

**Fig. 5:**
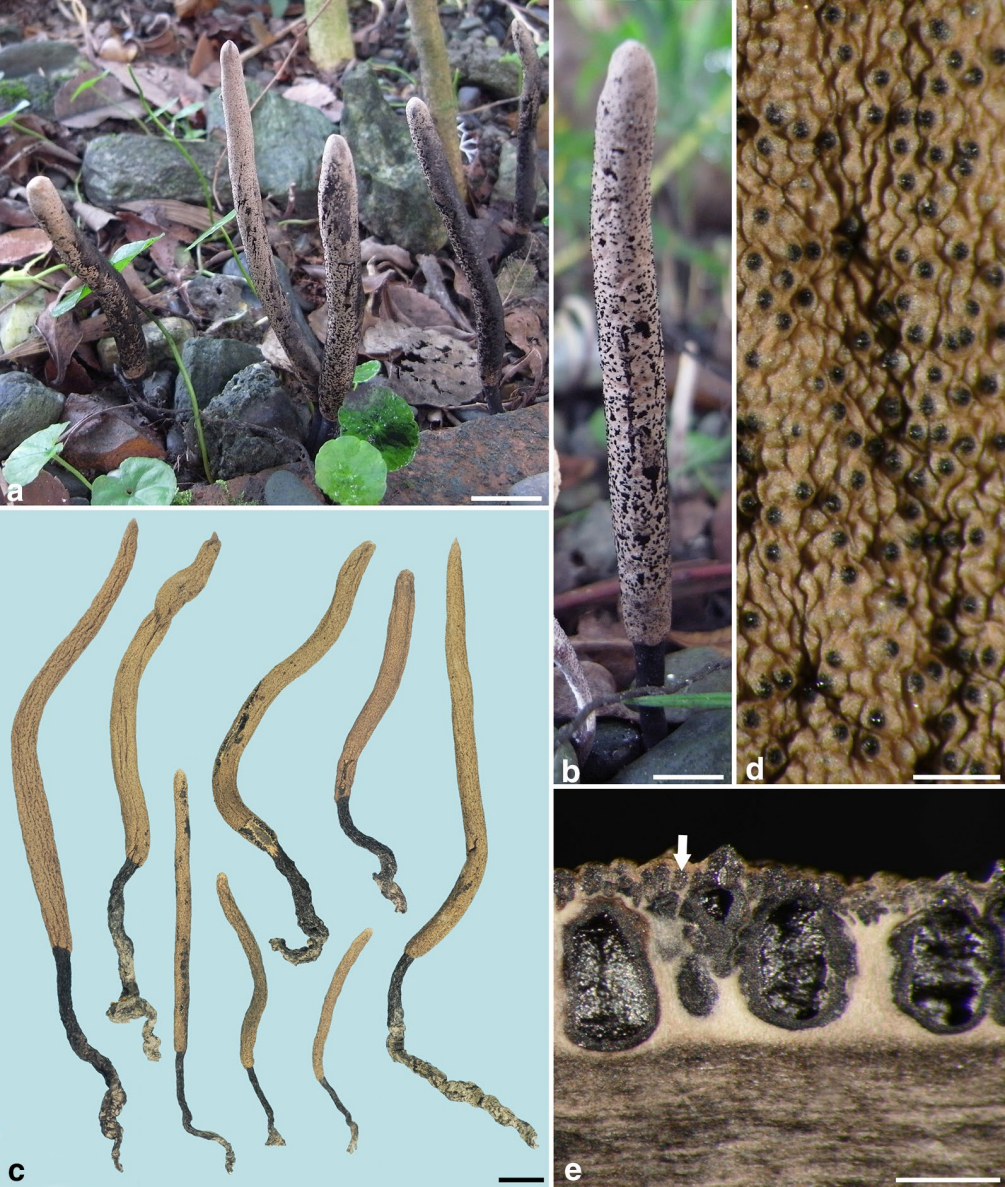
*Xylaria subescharoidea* (from the holotype). **a**, **b** Stromata in natural habit showing black exudated ascospore masses deposited on the surface. **c** Dried stromata. **d** Stromatal surface tuberculate at and between perithecial mounds. **e** Vertical section of perithecia; the arrow points towards one of the black ellipsoidal granules between ostioles. Bars in **a**, **c** = 1 cm; **b** = 0.5 cm; **d**, **e** = 0.5 mm

**Fig. 6:**
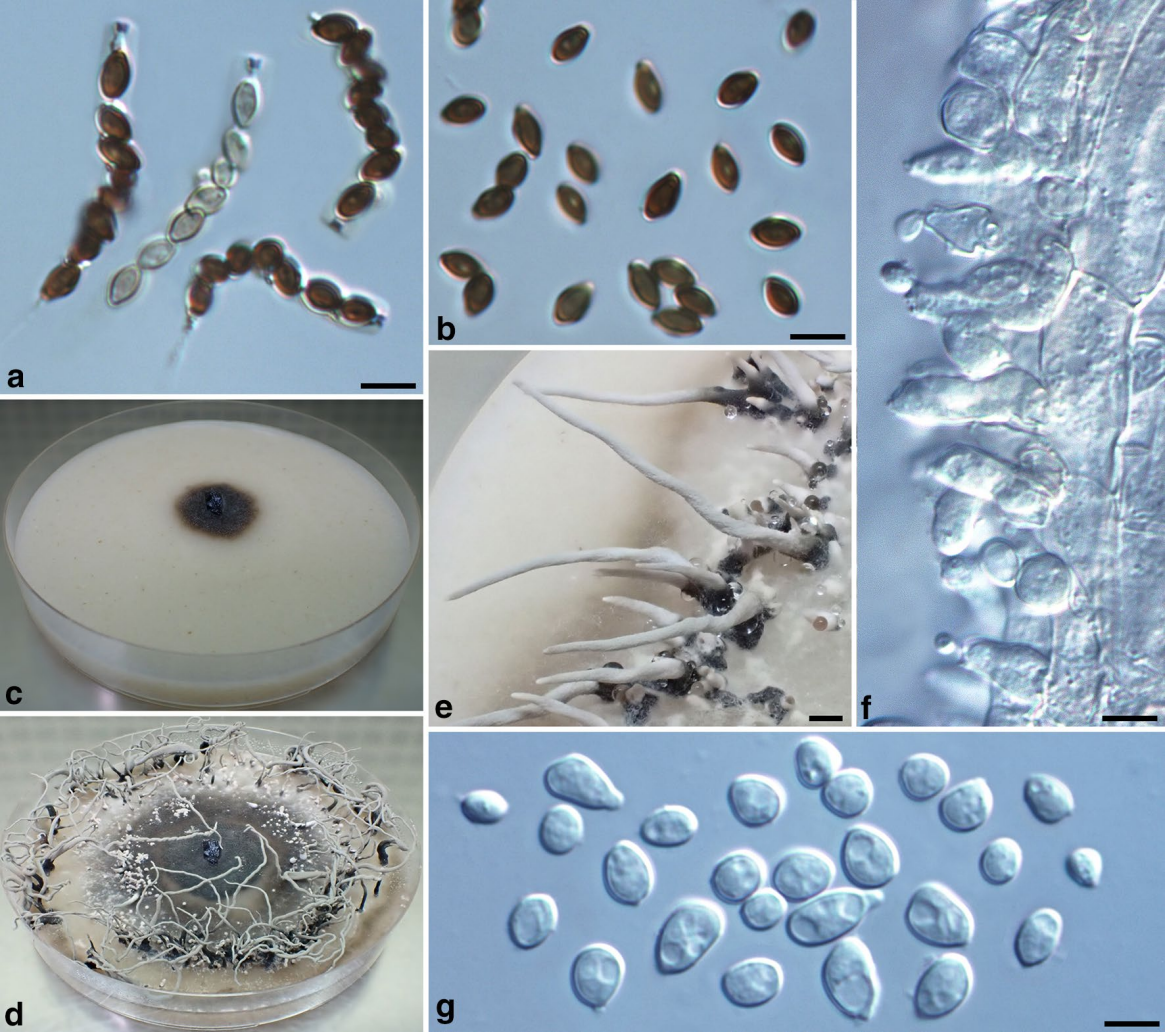
*Xylaria subescharoidea* (from the holotype). **a** Ascal apical rings and ascospores. **b** Ascospores with a half of these showing a pore-like germination site. **c**, **d** Colony on 9-cm Petri plate containing OA at 1.5 week and 3 week, respectively. **e** Stromata produced in culture. **f** Conidiophores. **g** Conidia. Bars in **a**, **b**, **f**, **g** = 5 μm; **e** = 2 mm

***Xylaria subescharoidea*** Y.-M. Ju, H.-M. Hsieh et J.-C. Chou, sp. nov. Figs. 5, 6.

MycoBank MB 834499.

**Etymology.** Referring to its stromata resembling those of *X. escharoidea* in gross morphology.

Stromata cylindrical to cylindric-fusoid at fertile part, unbranched, with a narrowly rounded to mucronate apex, on a long, glabrous stipe, with a tortuous rooting base, 4.5–11.5 cm long above ground, 3.5–9.5 cm long × 3–6 mm diam at fertile part; surface pale brown to ochraceous when fully mature, with conspicuous perithecial mounds and tuberculate between perithecial mounds, lacking an outer layer, underlain with a layer of black ellipsoidal granules between ostioles; interior white, hard, brittle, with a black core. Perithecia obovoid, 300–500 µm diam × 600–800 µm high. Ostioles papillate, ca. 100 µm broad at base. Asci with eight ascospores arranged in uniseriate manner, cylindrical, 50–65 µm total length, the spore-bearing part 25–33 µm long × 3–4 µm broad, with an apical ring staining blue in Melzer’s iodine reagent, inverted hat-shaped, 1–1.5 µm high × 1–1.5 µm broad. Ascospores brown to dark brown, unicellular, ellipsoid, nearly equilateral, with narrowly rounded ends, smooth, (4.0–)4.3–4.7 (–4.9) ×  (2.3–)2.5–2.9 (–3.0) µm (4.5 ± 0.2 × 2.7 ± 0.2 µm, N = 40), with a median, pore-like germination site, lacking a hyaline sheath; epispore smooth.

**Cultures and anamorph.** Colonies reaching the edge of 9-cm Petri dish in 3 week, whitish, immediately becoming blackish, mostly submerged, faintly zonate, with diffuse margins. Reverse uncolored. Stromata arising from concentric zones, cylindrical, tapering upwards, flexuous, unbranched, up to 4 cm long × 1.2–2.2 mm diam, black at base, white on the surface of upper part but becoming pale mouse gray due to production of conidia. Conidiophores composed of upright conidiogenous cells only. Conidiogenous cells arising directly from stromatal surface, cylindrical, 8.5–17 × 3.5–5 µm, smooth, bearing one to several terminal denticulate conidial secession scars. Conidia produced holoblastically in sympodial sequence, hyaline, smooth, variable in shape, subglobose, obovoid to ellipsoid, equilateral or slightly to significantly oblique, (4.3–)5.0–7.2 (–8.9) ×  (3.3–)3.7–4.5 (–4.7) µm (6.1 ± 1.1 × 4.1 ± 0.4 µm, N = 40), with a minute flattened base indicating former point of attachment to conidiogenous cell.

**Typification.** TAIWAN. Hua-lien County, Ji-an Township, Fu-hsin Village, from termite nests underground, 4 Jun–14 Jul 2010, *Chou, J.*-*C. 99060401* (cultured) (holotype HAST 144971), GenBank accessions: ITS = MN655980, *rpb2* = MN656982, *β*-*tub* = MN656984, *α*-*act* = MN656986.

**Additional specimen examined.** Tainan City, Nan-hsi District, on ground of mango orchard, 23 May 2006, *Chou, K.*-*H. 95052301* (cultured), as *X*. sp. 2 in Hsieh et al. (2010) (HAST), immature, GenBank accessions: ITS = GU324754, *rpb2* = GQ853025, *β*-*tub* = GQ502708, *α*-*act* = GQ853043.

**Notes.***Xylaria subescharoidea* is characterized by having pale brown to ochraceous, long cylindrical stromata, lacking an outer stromatal layer, lacking a continuous black layer immediately beneath the surface, having nearly equilateral ascospores that possess a pore-like germination site. Black ellipsoidal granules between ostioles form a layer below the surface and give rise to the tuberculate appearance of the stromatal surface. It remains unknown as to whether these granules possess certain functions or represent aborted perithecia. *Xylaria subescharoidea* is closely related to *X*. *escharoidea* (Fig. 2), with which it shares a pore-like ascospore germination site and long cylindrical stromata that possess a dark core and lack an outer layer. *Xylaria escharoidea* differs from *X*. *subescharoidea* by strongly inequilateral ascospores that are laterally compressed, a dark gray to dull black surface when fully mature, and a continuous black layer beneath the surface.

Conidiophores in most *Xylaria* species are dichotomously branched several times and have the conidiogenous cells densely arranged in palisades. The conidiophores of *X*. *subescharoidea*, however, are highly reduced to mostly upright conidiogenous cells, which are loosely arranged. This sets a difference between *X*. *subescharoidea* and *X*. *escharoidea*. The difference between the two species also lies in their colony growth rates, with the colonies of *X*. *escharoidea* covering 9-cm Petri dishes in 5 days, much faster than those of *X*. *subescharoidea*.

*Xylaria* sp. 2 in Hsieh et al. (2010) is based on an immature specimen, which is proven the same as the present species by culture morphology, the anamorph, and DNA sequences.

## Conclusion

Twelve out of 28 species of *Xylaria* known in the world have been recorded from termite nests or ground in Taiwan, with *X*. *furcata*, *X. insolita*, and *X. subescharoidea* included. *Xylaria* species growing on termite nests are primarily associated with macrotermitine termites, and *Odontotermes formosanus* is the only macrotermitine species known in Taiwan (Hsieh et al. 2010). Given the fact that the species number of macrotermitine termites in the world is approximately 330 (Kambhampati and Eggleton 2000), the global *Xylaria* diversity associated with termite nests is likely severely underestimated.

## Supplementary information

**Additional file 1.** List of taxa included in the present study.

**Additional file 2.** Overall tree topology resulting from BA analysis.

## Data Availability

Specimens are deposited at the herbarium HAST. Cultures are available at BCRC. DNA sequences are deposited at GenBank. Collecting data and GenBank accession numbers of the 135 isolates of 117 taxa included in the phylogenetic analyses are tabulated in Additional file 1. Overall tree topology resulting from BA can be found in Additional file 2.
