# Supplementary material for: Xylaria insolita and X. subescharoidea: two newly described species collected from a termite nesting site in Hua-lien, Taiwan
Source: Bot Stud. 2020 Apr 6;61:11. doi: 10.1186/s40529-020-00287-1 (PMC7136384; doi:10.1186/s40529-020-00287-1)
Supplement: Supplementary file 1 — Additional file 1. List of taxa included in the present study. [file 40529_2020_287_MOESM1_ESM.doc]

Table S1. Taxa included in the present study. Note that sequences of those taxa in boldface were generated in this study.

| Taxon | Origin | Collecting data | GenBank accession number | | |
| --- | --- | --- | --- | --- | --- |
|  |  |  | β-tubulin gene | α-actin gene | RPB2 gene |
| *Amphirosellinia fushanensis* Y.-M. Ju et al. | Taiwan | HOLOTYPE (Ju et al. 2004) | GQ495950 | GQ452360 | GQ848339 |
| *Amphirosellinia nigrospora* Y.-M. Ju et al. | Taiwan | HOLOTYPE (Ju et al. 2004) | GQ495951 | GQ452361 | GQ848340 |
| *Annulohypoxylon cohaerens* (Pers.) Y.-M. Ju et al. | France | *Fournier 03041* (Hsieh et al. 2005) | AY951655 | AY951766 | GQ844766 |
| *Astrocystis bambusae* (Henn.) Læssøe & Spooner | Taiwan | *Ju & Hsieh 89021904* (Hsieh et al. 2010) | GQ495942 | GQ449239 | GQ844836 |
| *Astrocystis mirabilis* Berk. & Broome | Taiwan | *Ju & Hsieh 94070803* (Hsieh et al. 2010) | GQ495941 | GQ449238 | GQ844835 |
| *Astrocystis sublimbata* (Durieu & Mont.) G. C. Hughes | Taiwan | *Ju & Hsieh 89032207* (Hsieh et al. 2010) | GQ495940 | GQ449236 | GQ844834 |
| *Biscogniauxia arima* San Martín et al. | Mexico | ISOTYPE (Hsieh et al. 2005; Ju et al. 1998) | AY951672 | AY951784 | GQ304736 |
| *Biscogniauxia mediterranea* (De Not.) Kuntze | France | *Candoussau 366* (Hsieh et al. 2005; Ju et al. 1998) | AY951684 | AY951796 | GQ844765 |
| *Discoxylaria myrmecophila* J. C. Lindq. & J. E. Wright | Mexico | *Moreno 713* (Rogers et al. 1995) | GQ487710 | GQ438747 | GQ844819 |
| *Entoleuca mammata* (Wahlenb.) J. D. Rogers & Y.-M. Ju | France | *Candoussau, F. 5254* (Hsieh et al. 2010) | GQ470230 | GQ398230 | GQ844782 |
| *Euepixylon sphaeriostomum* (Schwein.) Y.-M. Ju & J. D. Rogers | USA | *Huhndorf, S. M. 1447* (Hsieh et al. 2010) | GQ470224 | GQ389696 | GQ844774 |
| *Kretzschmaria clavus* (Fr.) Sacc. | French Guiana | *Huhndorf 803* (Ju et al. 2007; Rogers and Ju 1998) | EF025611 | EF025596 | GQ844789 |
| *Kretzschmaria guyanensis* J. D. Rogers & Y.-M. Ju | Taiwan | *Ju & Hsieh 89062903* (Hsieh et al. 2010) | GQ478214 | GQ408901 | GQ844792 |
| *Kretzschmaria lucidula* (Mont.) Dennis | French Guiana | *Huhndorf 677* (Ju et al. 2007; Rogers and Ju 1998) | EF025610 | EF025595 | GQ844790 |
| *Kretzschmaria megalospora* J. D. Rogers & Y.-M. Ju | Malaysia | *Whalley, M. FH 64-97* (Ju et al. 2007) | EF025609 | EF025594 | GQ844791 |
| *Kretzschmaria neocaledonica* (Har. & Pat.) J. D. Rogers & Y.-M. Ju | Taiwan | *Guu, J.-R. 94031003* (Hsieh et al. 2010) | GQ478213 | GQ398236 | GQ844788 |
| *Kretzschmaria pavimentosa* (Ces.) P. Martin | Taiwan | *Wang 511* (Rogers and Ju 1998) | GQ478212 | GQ398235 | GQ844787 |
| *Kretzschmaria sandvicensis* (Reichardt) J. D. Rogers & Y.-M. Ju | USA, Hawaiian Islands | *Rogers/4 Jan 1996* (Rogers and Ju 1998) | GQ478211 | GQ398234 | GQ844786 |
| *Nemania abortiva* J. D. Rogers et al. | USA, Hawaiian Islands | HOLOTYPE (Rogers et al. 2006) | GQ470219 | GQ374123 | GQ844768 |
| *Nemania beaumontii* (Berk. & M. A. Curtis) Y.-M. Ju & J. D. Rogers | French West Indies | *Lechat, C. CLL2039* (Fournier et al. 2018a; Hsieh et al. 2010) | GQ470222 | GQ389694 | GQ844772 |
| *Nemania bipapillata* (Berk. & M. A. Curtis) Pouzar | Taiwan | *Ju & Hsieh 90080610* (Hsieh et al. 2010) | GQ470221 | GQ389693 | GQ844771 |
| *Nemania diffusa* (Sowerby) S. F. Gray | Taiwan | *Ju & Hsieh 91020401* (Hsieh et al. 2010) | GQ470220 | GQ389692 | GQ844769 |
| *Nemania illita* (Schwein.) Pouzar | USA | *Tsai, S.-J.* (Ju et al. 2007) | EF025608 | EF025593 | GQ844770 |
| *Nemania macrocarpa* Y.-M. Ju & J. D. Rogers | USA, Hawaiian Islands | HOLOTYPE (Ju and Rogers 2002) | GQ470226 | GQ389698 | GQ844776 |
| *Nemania maritima* Y.-M. Ju & J. D. Rogers | Taiwan | HOLOTYPE (Ju and Rogers 2002) | GQ470225 | GQ389697 | GQ844775 |
| *Nemania primolutea* Y.-M. Ju et al. | Taiwan | HOLOTYPE (Ju et al. 2007; Ju et al. 2005) | EF025607 | EF025592 | GQ844767 |
| *Nemania serpens* (Pers.) S. F. Gray *“*Barron isolate” | Canada | *Barron, G.*, as *Hypoxylon* in Petrini and Rogers (1986) | GQ470223 | GQ389695 | GQ844773 |
| *Podosordaria mexicana* Ellis & Holw. | Mexico | *San Martín 6013T* (Rogers et al. 1998) | GQ844840 | GQ455451 | GQ853039 |
| *Podosordaria muli* J. D. Rogers et al. | Mexico | HOLOTYPE (Rogers et al. 1998) | GQ844839 | GQ455450 | GQ853038 |
| *Poronia pileiformis* (Berk.) Fr. | Taiwan | EPITYPE (Ju and Rogers 2001) | GQ502720 | GQ455449 | GQ853037 |
| *Rosellinia buxi* Fabre | France | *Candoussau, F.* (Hsieh et al. 2010) | GQ470228 | GQ398228 | GQ844780 |
| *Rosellinia lamprostoma* Syd. & P. Syd. | Taiwan | *Ju & Hsieh 89112602* (Ju et al. 2007) | EF025604 | EF025589 | GQ844778 |
| *Rosellinia merrillii* Syd. & P. Syd. | Taiwan | *Ju & Hsieh 89112601* (Hsieh et al. 2010) | GQ470229 | GQ398229 | GQ844781 |
| *Rosellinia necatrix* (R. Hartig) Berl. | Taiwan | *Ju & Hsieh 89062904* (Ju et al. 2007) | EF025603 | EF025588 | GQ844779 |
| *Rosellinia sanctaecruciana* Ferd. & Winge | Taiwan | *Ju & Hsieh 90072903* (Hsieh et al. 2010) | GQ470227 | GQ389699 | GQ844777 |
| *Stilbohypoxylon elaeicola* (Henn.) L. E. Petrini | French Guiana | *Huhndorf 928* (Ju et al. 2007), as *S. moelleri* in Rogers and Ju (1997) | EF025616 | EF025601 | GQ844826 |
| *Stilbohypoxylon elaeicola* (Henn.) L. E. Petrini | Taiwan | *Ju & Hsieh 94082615* (Hsieh et al. 2010) | GQ495933 | GQ438754 | GQ844827 |
| *Stilbohypoxylon quisquiliarum* (Mont.) J. D. Rogers & Y.-M. Ju | French Guiana | *Huhndorf 940* (Ju et al. 2007; Rogers and Ju 1997) | EF025605 | EF025590 | GQ853020 |
| *Stilbohypoxylon quisquiliarum* (Mont.) J. D. Rogers & Y.-M. Ju | Taiwan | *Ju & Hsieh 89091608* (Ju et al. 2007) | EF025606 | EF025591 | GQ853021 |
| *Xylaria acuminatilongissima* Y.-M. Ju & H.-M. Hsieh | Taiwan | HOLOTYPE (Ju and Hsieh 2007) | GQ502711 | GQ853046 | GQ853028 |
| *Xylaria adscendens* (Fr.) Fr. | French West Indies | *Lechat, C. CLL5347* (Hsieh et al. 2010) | GQ487708 | GQ438745 | GQ844817 |
| *Xylaria adscendens* (Fr.) Fr. | Thailand | *Bandoni, R. J., Bandoni, A. A. & Flegel, T. W. 12017* (Hsieh et al. 2010) | GQ487709 | GQ438746 | GQ844818 |
| *Xylaria alboareolata Y.-M. Ju & J.D. Rogers* | French West Indies | *Chabrol, J. CLL5372 (Fournier et al. 2018b), as X. areolata in Hsieh et al. (2010)* | GQ478215 | GQ408902 | GQ844793 |
| *Xylaria allantoidea* (Berk.) Fr. | Taiwan | *Ju & Hsieh 94042903* (Hsieh et al. 2010) | GQ502692 | GQ452377 | GQ848356 |
| *Xylaria amphithele* San Martín & J. D. Rogers | French West Indies | *Lechat, C. CLL5352* (Hsieh et al. 2010) | GQ478218 | GQ408905 | GQ844796 |
| *Xylaria apoda* (Berk. & Broome) J. D. Rogers & Y.-M. Ju | Taiwan | *Ju & Hsieh 90080804* (Hsieh et al. 2010) | GQ495930 | GQ438751 | GQ844823 |
| *Xylaria arbuscula* Sacc. | Taiwan | *Ju & Hsieh 89041211* (Hsieh et al. 2010) | GQ478226 | GQ421286 | GQ844805 |
| *Xylaria arbuscula* var. *plenofissura* Y.-M. Ju & S.-S. Tzean | Taiwan | *Ju & Hsieh 93082814* (Hsieh et al. 2010) | GQ478225 | GQ421285 | GQ844804 |
| *Xylaria atrodivaricata* Y.-M. Ju & H.-M. Hsieh | Taiwan | HOLOTYPE (Ju and Hsieh 2007) | GQ502713 | GQ853048 | GQ853030 |
| *Xylaria atrosphaerica* (Cooke & Massee) Callan & J. D. Rogers | Taiwan | *Ju & Hsieh 91111214* (Hsieh et al. 2010) | GQ495953 | GQ452363 | GQ848342 |
| *Xylaria badia* Pat. | Taiwan | *Ju & Hsieh 95070101* (Hsieh et al. 2010) | GQ495939 | GQ449235 | GQ844833 |
| *Xylaria bambusicola* Y.-M. Ju & J. D. Rogers | Taiwan | HOLOTYPE (Hsieh et al. 2005; Ju and Rogers 1999) | AY951762 | AY951873 | GQ844802 |
| *Xylaria bambusicola* Y.-M. Ju & J. D. Rogers | Thailand | *Bandoni, R. J. & A. A. et al.* (Hsieh et al. 2010) | GQ478223 | GQ408910 | GQ844801 |
| *Xylaria berteri* (Mont.) Cooke | USA, Hawaiian Islands | *Rogers, J. D. K-1* (Hsieh et al. 2010) | GQ502698 | GQ455442 | GQ848363 |
| *Xylaria berteri* (Mont.) Cooke | Taiwan | *Ju & Hsieh 90112623* (Hsieh et al. 2005) | AY951763 | AY951874 | GQ848362 |
| *Xylaria brunneovinosa* Y.-M. Ju & H.-M. Hsieh | Taiwan | HOLOTYPE (Ju and Hsieh 2007) | GQ502706 | GQ853041 | GQ853023 |
| *Xylaria cantareirensis* (Henn.) J. Fourn. & Lechat | French West Indies | Lechat, C. CLL5437 (Fournier et al. 2018b), as Penzigia in Hsieh et al. (2010) | GQ478220 | GQ408907 | GQ844798 |
| *Xylaria castorea* Berk. | New Zealand | *Samuels 85-75* (Hsieh et al. 2010) | GQ502703 | GQ455447 | GQ853018 |
| *Xylaria* cf. *castorea* Berk. | Taiwan | *Ju & Hsieh 91092303* (Hsieh et al. 2010) | GQ502704 | GQ455448 | GQ853019 |
| *Xylaria cirrata* Pat. | Taiwan | EPITYPE (Ju and Hsieh 2007) | GQ502707 | GQ853042 | GQ853024 |
| *Xylaria coccophora* Mont. | French Guiana | *Lechat, C. CLL7056* (Hsieh et al. 2010) | GQ487701 | GQ421289 | GQ844809 |
| *Xylaria coprinicola* Y.-M. Ju et al. | China | HOLOTYPE (Ju et al. 2011) | HM585018 | HM585017 | HM585019 |
| *Xylaria cranioides* (Sacc. & Paol.) Dennis | Taiwan | *Wen 712* (Ju and Rogers 2001) | GQ478210 | GQ398233 | GQ844785 |
| *Xylaria crozonensis* P. Leroy & Mornand | France | *Mornand, F. JF04151* (Hsieh et al. 2010) | GQ502697 | GQ455441 | GQ848361 |
| *Xylaria cubensis* (Mont.) Fr. | French West Indies | *Lechat, C. CLL2179* (Fournier et al. 2019), as *X*. *laevis* in Hsieh et al. (2010) | GQ502695 | GQ455439 | GQ848359 |
| *Xylaria cubensis* (Mont.) Fr. | Taiwan | *Ju & Hsieh 95072910*, as *X*. *laevis* in Hsieh et al. (2010) | GQ502696 | GQ455440 | GQ848360 |
| *Xylaria culleniae* Berk. & Broome | Thailand | *Whalley, M. F.NH9* (Hsieh et al. 2010) | GQ495935 | GQ438756 | GQ844829 |
| *Xylaria cuneata* C. G. Lloyd | French West Indies | *Lechat, C. CLL5131* (Fournier et al. 2019)*,* as *X*. *montagnei* Hamme & Guerrero in Hsieh et al. (2010) | GQ495948 | GQ449244 | GQ848337 |
| *Xylaria curta* Fr. | French West Indies | *Lechat, C. CLL5044* (Hsieh et al. 2010) | GQ495937 | GQ449233 | GQ844831 |
| *Xylaria curta* Fr. | Taiwan | *Ju & Hsieh 92092022* (Hsieh et al. 2010) | GQ495936 | GQ438757 | GQ844830 |
| *Xylaria digitata* (L.) Grev. | Ukraine | *Prilutsky, O.* (Hsieh et al. 2010) | GQ495949 | GQ449245 | GQ848338 |
| *Xylaria enterogena* (Mont.) Fr. | French Guiana | *Lechat, C. CLL7043* (Hsieh et al. 2010) | GQ502685 | GQ452370 | GQ848349 |
| *Xylaria escharoidea* (Berk.) Fr. | Taiwan | EPITYPE (Ju and Hsieh 2007) | GQ502709 | GQ853044 | GQ853026 |
| *Xylaria feejeensis* (Berk.) Fr. | French West Indies | *Lechat, C. CLL5653* (Hsieh et al. 2010) | GQ495945 | GQ449241 | GQ848334 |
| *Xylaria feejeensis* (Berk.) Fr. | Taiwan | *Ju & Hsieh 92092013* (Hsieh et al. 2010) | GQ495947 | GQ449243 | GQ848336 |
| *Xylaria feejeensis* (Berk.) Fr. | Thailand | *Whalley, M. UN515* (Hsieh et al. 2010) | GQ495946 | GQ449242 | GQ848335 |
| *Xylaria fimbriata* C. G. Lloyd | French West Indies | *Lechat, C. CLL5010* (Hsieh et al. 2010) | GQ502705 | GQ853040 | GQ853022 |
| *Xylaria fissilis* Ces. | French West Indies | *Lechat, C. CLL0928* (Hsieh et al. 2010) | GQ470231 | GQ398231 | GQ844783 |
| *Xylaria flabelliformis* (Schwein.) Fr. | USA | *Rogers, J. D.*, as *X*. *cubensis* in Hsieh et al. (2010) | GQ502700 | GQ455444 | GQ848365 |
| *Xylaria flabelliformis* (Schwein.) Fr. | Papua New Guinea | *Van der Gucht & De Meester 92-521* (Hsieh et al. 2010; Van der Gucht 1995), as *X*. *cubensis* | GQ502702 | GQ455446 | GQ853017 |
| *Xylaria flabelliformis* (Schwein.) Fr. | Russian Far East | *Vasilyeva, L. N.*, as *X*. *cubensis* in Hsieh et al. (2010) | GQ502699 | GQ455443 | GQ848364 |
| *Xylaria flabelliformis* (Schwein.) Fr. | French West Indies | *Lechat, C. CLL5121* (Fournier et al. 2019), as *X*. *cubensis* in Hsieh et al. (2010) | GQ502701 | GQ455445 | GQ848366 |
| *Xylaria frustulosa* (Berk. & M. A. Curtis) Cooke | French West Indies | *Lechat, C. CLL6002-2* (Fournier et al. 2018b; Hsieh et al. 2010) | GQ495943 | GQ449237 | GQ844837 |
| *Xylaria frustulosa* (Berk. & M. A. Curtis) Cooke | Taiwan | *Ju & Hsieh 92092010* (Hsieh et al. 2010) | GQ495944 | GQ449240 | GQ844838 |
| *Xylaria rhytidosperma* J. Fourn. & Lechat | French West Indies | ISOTYPE (Fournier et al. 2018b), as *X*. cf. *glebulosa* in Hsieh et al. (2010) | GQ495956 | GQ452366 | GQ848345 |
| *Xylaria globosa* (Spreng. ex Fr.) Mont. | French West Indies | *Lechat, C. CLL6033* (Hsieh et al. 2010) | GQ502684 | GQ452369 | GQ848348 |
| *Xylaria grammica* (Mont.) Fr. | Taiwan | *Chen, G.-T.* (Hsieh et al. 2010) | GQ487704 | GQ427197 | GQ844813 |
| *Xylaria griseosepiacea* Y.-M. Ju & H.-M. Hsieh | Taiwan | HOLOTYPE (Ju and Hsieh 2007) | GQ502714 | GQ853049 | GQ853031 |
| *Xylaria haemorrhoidalis* Berk. & Broome | Taiwan | *Ju & Hsieh 89041207* (Hsieh et al. 2010) | GQ502683 | GQ452368 | GQ848347 |
| *Xylaria* cf. *heliscus* (Mont.) J. D. Rogers & Y.-M. Ju | Taiwan | *Ju & Hsieh 88113010* (Hsieh et al. 2010) | GQ502691 | GQ452376 | GQ848355 |
| *Xylaria hypoxylon* (L.) Grev. | Belgium | *Ju, Y.-M.* (Hsieh et al. 2010) | GQ260187 | GQ427196 | GQ844812 |
| *Xylaria hypoxylon* (L.) Grev. | Taiwan | *Guu, J.-R. 95082001* (Hsieh et al. 2010) | GQ487703 | GQ427195 | GQ844811 |
| *Xylaria ianthinovelutina* (Mont.) Fr. | French West Indies | *Lechat, C. CLL5599* (Hsieh et al. 2010) | GQ495934 | GQ438755 | GQ844828 |
| ***Xylaria insolita*** Y.-M. Ju et al. | Taiwan | HOLOTYPE (the present study) | **MN656983** | **MN656985** | **MN656981** |
| *Xylaria intracolorata* (J. D. Rogers et al.) J. D. Rogers & Y.-M. Ju | Taiwan | *Ju & Hsieh 90080402* (Hsieh et al. 2010) | GQ502690 | GQ452375 | GQ848354 |
| *Xylaria intraflava* Y.-M. Ju & H.-M. Hsieh | Taiwan | HOLOTYPE (Ju and Hsieh 2007) | GQ502718 | GQ853053 | GQ853035 |
| *Xylaria juruensis* Henn. | Taiwan | *Ju & Hsieh 92042501* (Hsieh et al. 2010) | GQ495932 | GQ438753 | GQ844825 |
| *Xylaria liquidambar* J. D. Rogers et al. | Taiwan | *Ju & Hsieh 93090701* (Hsieh et al. 2010) | GQ487702 | GQ421290 | GQ844810 |
| *Xylaria luteostromata* C. G. Lloyd var*. macrospora* J. D. Rogers & Samuels | French West Indies | *Lechat, C. CLL5020* (Hsieh et al. 2010) | GQ502688 | GQ452373 | GQ848352 |
| *Xylaria meliacearum* Læssøe | Puerto Rico | *Lodge, D. J. PR-894* (Læssøe and Lodge 1994) | GQ478219 | GQ408906 | GQ844797 |
| *Xylaria microceras* (Mont.) Fr. | French West Indies | *Lechat, C. CLL2265* (Hsieh et al. 2010) | GQ478221 | GQ408908 | GQ844799 |
| *Xylaria multiplex* (Kunze) Fr. | French West Indies | *Lechat, C. CLL5287* (Hsieh et al. 2010) | GQ487705 | GQ427198 | GQ844814 |
| *Xylaria multiplex* (Kunze) Fr. | USA, Hawaiian Islands | *Hemmes, D. E. Xy-7* (Hsieh et al. 2010) | GQ487706 | GQ438743 | GQ844815 |
| *Xylaria muscula* C. G. Lloyd | French West Indies | *Lurel, D. CLL5323* (Hsieh et al. 2010) | GQ478222 | GQ408909 | GQ844800 |
| *Xylaria nigripes* (Klotzsch) Fr. | Taiwan | *Chou, K.-H. 94053001* (Ju and Hsieh 2007) | GQ502710 | GQ853045 | GQ853027 |
| *Xylaria ochraceostroma* Y.-M. Ju & H.-M. Hsieh | Taiwan | HOLOTYPE (Ju and Hsieh 2007) | GQ502717 | GQ853052 | GQ853034 |
| *Xylaria oligotoma* Sacc. & Paol. | French Guiana | *Lechat, C. CLL7031* (Hsieh et al. 2010) | GQ487700 | GQ421288 | GQ844808 |
| *Xylaria ophiopoda* Sacc. | Taiwan | *Ju & Hsieh 93082805* (Hsieh et al. 2010) | GQ495955 | GQ452365 | GQ848344 |
| *Xylaria oxyacanthae* Tul. & C. Tul. | USA | *Yeomans, R.* (Hsieh et al. 2010) | GQ495927 | GQ438748 | GQ844820 |
| *Xylaria palmicola* G. Winter | New Zealand | *Samuels, G. J. 85-83* (Hsieh et al. 2010) | GQ495929 | GQ438750 | GQ844822 |
| *Xylaria papulis* C. G. Lloyd | Taiwan | *Ju & Hsieh 89021903* (Hsieh et al. 2010) | GQ487707 | GQ438744 | GQ844816 |
| *Xylaria* *phyllocharis* Mont. | French West Indies | *Lechat, C. CLL5302* (Hsieh et al. 2010) | GQ495938 | GQ449234 | GQ844832 |
| *Xylaria plebeja* Ces. | Taiwan | *Ju & Hsieh 91122401* (Hsieh et al. 2010) | GQ502689 | GQ452374 | GQ848353 |
| *Xylaria polymorpha* (Pers.) Grev. | USA | *Rogers, J. D.* (Hsieh et al. 2010) | GQ495954 | GQ452364 | GQ848343 |
| *Xylaria reevesiae* Y.-M. Ju et al. | Taiwan | HOLOTYPE (Ju et al. 2018), as *X*. sp. 7 in Hsieh et al. (2010) | GQ495928 | GQ438749 | GQ844821 |
| *Xylaria regalis* Cooke | India | *Gailawad, S. AMH 9204* (Hsieh et al. 2010) | GQ502694 | GQ452379 | GQ848358 |
| *Xylaria regalis* Cooke | Taiwan | *Ju & Hsieh 92072001* (Hsieh et al. 2010) | GQ502693 | GQ452378 | GQ848357 |
| *Xylaria schweinitzii* Berk. & M. A. Curtis | Taiwan | *Ju & Hsieh 92092023* (Hsieh et al. 2010) | GQ495957 | GQ452367 | GQ848346 |
| *Xylaria scruposa* (Fr.) Fr. | French West Indies | *Lechat, C. CLL5025* (Hsieh et al. 2010) | GQ495952 | GQ452362 | GQ848341 |
| *Xylaria sicula* Pass. & Beltr. f. *major* Ciccarone | Taiwan | *Ju & Hsieh 90071613* (Hsieh et al. 2010) | GQ478216 | GQ408903 | GQ844794 |
| *Xylaria* sp. 1 (from termite nests) | Taiwan | *Ju & Hsieh 95052006* (Hsieh et al. 2010) | GQ502719 | GQ853054 | GQ853036 |
| *Xylaria* sp. 3 (from termite nests) | Taiwan | *Chou, K.-H. 95060503* (Hsieh et al. 2010) | GQ502712 | GQ853047 | GQ853029 |
| *Xylaria* sp. 4 (from termite nests) | Taiwan | *Chou, K.-H. 95072001* (Hsieh et al. 2010) | GQ502715 | GQ853050 | GQ853032 |
| *Xylaria* sp. 5 (from termite nests) | Taiwan | *Chou, K.-H. 95071201* (Hsieh et al. 2010) | GQ502716 | GQ853051 | GQ853033 |
| *Xylaria* sp. 6 (from leaves) | USA, Hawaiian Islands | *Hemmes, D. E. DEH-1052* (Hsieh et al. 2010) | GQ478217 | GQ408904 | GQ844795 |
| *Xylaria striata* Pat. | China | *Leu, L.-S.* (Hsieh et al. 2010) | GQ478224 | GQ421284 | GQ844803 |
| ***Xylaria subescharoidea*** Y.-M. Ju et al. | Taiwan | HOLOTYPE (the present study) | MN656984 | MN656986 | MN656982 |
| ***Xylaria subescharoidea*** Y.-M. Ju et al. | Taiwan | *Chou, K.-H. 95052301*, as *X*. sp. 2 in Hsieh et al. (2010); immature | GQ502708 | GQ853043 | GQ853025 |
| *Xylaria telfairii* (Berk.) Fr. | French West Indies | *Lechat, C. CLL2224* (Fournier et al. 2019; Hsieh et al. 2010) | GQ502686 | GQ452371 | GQ848350 |
| *Xylaria telfairii* (Berk.) Fr. | Taiwan | *Ju & Hsieh 90081901* (Hsieh et al. 2010) | GQ502687 | GQ452372 | GQ848351 |
| *Xylaria terricola* Y.-M. Ju et al. | Taiwan | HOLOTYPE (Chou et al. 2017) | MF577044 | MF577045 | MF577043 |
| *Xylaria tuberoides* Rehm | French West Indies | *Lechat, C. CLL2146* (Fournier et al. 2019; Hsieh et al. 2010) | GQ478209 | GQ398232 | GQ844784 |
| *Xylaria venosula* Speg. | USA, Hawaiian Islands | *Ju & Hsieh 94080508* (Ju et al. 2007) | EF025617 | EF025602 | GQ844806 |
| *Xylaria venustula* Sacc. | Taiwan | *Ju & Hsieh 88113002* (Hsieh et al. 2010) | GQ487699 | GQ421287 | GQ844807 |
| *Xylaria* *vivantii* Y.-M. Ju et al. | French West Indies | HOLOTYPE (Ju et al. 2018), as *X*. sp. 8 in Hsieh et al. (2010) | GQ495931 | GQ438752 | GQ844824 |

References

Chou W-N, Hsieh H-M, Ju Y-M. 2017. *Xylaria terricola* sp. nov., a terrestrial anamorphic *Xylaria* species found in Taiwan. Fungal Science 32:1–8.

Fournier J, Lechat C, Courtecuisse R. 2018a. The genera *Kretzschmariella* and *Nemania* (Xylariaceae) in Guadeloupe and Martinique (French West Indies). Ascomycete.org 10 1–47.

Fournier J, Lechat C, Courtecuisse R. 2018b. The genus *Xylaria* sensu lato (Xylariaceae) in Guadeloupe and Martinique (French West Indies) I. Taxa with penzigioid stromata. Ascomycete.org 10 131–176.

Fournier J, Lechat C, Courtecuisse R. 2019. The genus *Xylaria* sensu lato (Xylariaceae) in Guadeloupe and Martinique (French West Indies) II. Taxa with robust upright stromata. Ascomycete.org 11 77–115.

Hsieh H-M, Ju Y-M, Rogers JD. 2005. Molecular phylogeny of *Hypoxylon* and closely related genera. Mycologia 97:844–865.

Hsieh H-M, Lin C-R, Fang M-J, Rogers JD, Fournier J, Lechat C, Ju Y-M. 2010. Phylogenetic status of *Xylaria* subgen. *Pseudoxylaria* among taxa of the subfamily Xylarioideae (Xylariaceae) and phylogeny of the taxa involved in the subfamily. Molecular Phylogenetics and Evolution 54:957–969.

Ju Y-M, Hsieh H-M. 2007. *Xylaria* species associated with nests of *Odontotermes formosanus* in Taiwan. Mycologia 99:936–957.

Ju Y-M, Hsieh H-M, He X-S. 2011. *Xylaria coprinicola*, a new species that antagonizes cultivation of *Coprinus comatus* in China. Mycologia 103:424–430.

Ju Y-M, Hsieh H-M, Ho M-C, Szu D-H, Fang M-J. 2007. *Theissenia rogersii* sp. nov. and phylogenetic position of *Theissenia*. Mycologia 99:612–621.

Ju Y-M, Rogers JD. 1999. The Xylariaceae of Taiwan (excluding *Anthostomella*). Mycotaxon 73:343–440.

Ju Y-M, Rogers JD. 2001. *Xylaria cranioides* and *Poronia pileiformis* and their anamorphs in culture, and implications for the status of *Penzigia*. Mycological research 105:1134–1136.

Ju Y-M, Rogers JD. 2002. The genus *Nemania*. Nova Hedwigia 74:75–120.

Ju Y-M, Rogers JD, Hsieh H-M. 2004. *Amphirosellinia* gen. nov. and a new species of *Entoleuca*. Mycologia 96:1393–1402.

Ju Y-M, Rogers JD, Hsieh H-M. 2005. New *Hypoxylon* and *Nemania* species from Costa Rica and Taiwan. Mycologia 97:562–567.

Ju Y-M, Rogers JD, Hsieh H-M. 2018. *Xylaria* species associated with fallen fruits and seeds. Mycologia 110:726–749.

Ju Y-M, Rogers JD, San Martin F, Granmo A. 1998. The genus *Biscogniauxia*. Mycotaxon 66:1–98.

Læssøe T, Lodge DJ. 1994. Three host specific *Xylaria* species. Mycologia 86:436–446.

Petrini L, Rogers JD. 1986. A summary of the *Hypoxylon serpens* complex. Mycotaxon 26:401–436.

Rogers JD, Ju Y-M. 1997. The genus *Stilbohypoxylon*. Mycological research 101:135–138.

Rogers JD, Ju Y-M. 1998. The genus *Kretzschmaria*. Mycotaxon 68:345–393.

Rogers JD, Ju Y-M, Hemmes DE. 2006. *Hypoxylon subdisciforme* sp. nov., *Nemania abortiva* sp. nov., and *Xylotumulus gibbisporus* gen. et sp. nov. from Hawaii, Hawaiian Islands. Sydowia 58:290–299.

Rogers JD, Ju Y-M, San Martin F. 1995. *Discoxylaria myrmecophila* and its *Hypocreodendron* anamorph. Mycologia 87:41–45.

Rogers JD, Ju Y-M, San Martín F. 1998. *Podosordaria*: a redefinition based on cultural studies of the type species, *P. mexicana*, and two new species. Mycotaxon 67:61–72.

Van der Gucht K. 1995. Illustrations and descriptions of xylariaceous fungi collected in Papua New Guinea. Bulletin du Jardin Botanique National de

Belgique 64:219–403.
