# Supplementary figures and images for: Xylaria insolita and X. subescharoidea: two newly described species collected from a termite nesting site in Hua-lien, Taiwan
Source: Bot Stud. 2020 Apr 6;61:11. doi: 10.1186/s40529-020-00287-1 (PMC7136384; doi:10.1186/s40529-020-00287-1)

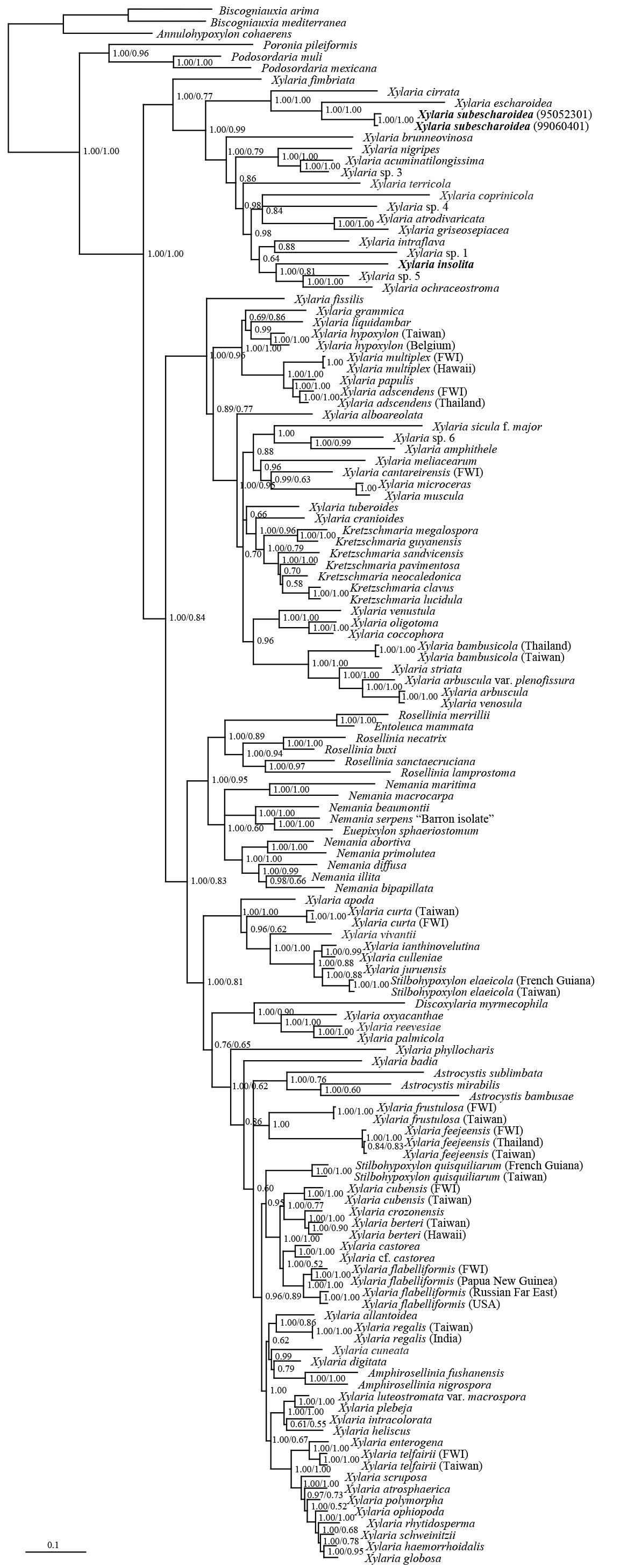

Supplement: Supplementary file 2 — Additional file 2. Overall tree topology resulting from BA analysis. [file 40529_2020_287_MOESM2_ESM.jpg]
